# Antibacterial Activity and Cytocompatibility of Bone Cement Enriched with Antibiotic, Nanosilver, and Nanocopper for Bone Regeneration

**DOI:** 10.3390/nano9081114

**Published:** 2019-08-03

**Authors:** Marcin Wekwejt, Anna Michno, Karolina Truchan, Anna Pałubicka, Beata Świeczko-Żurek, Anna Maria Osyczka, Andrzej Zieliński

**Affiliations:** 1 Biomaterials Division, Department of Materials Engineering and Bonding, Gdańsk University of Technology, 80-233 Gdańsk, Poland; 2 Chair of Clinical Biochemistry, Department of Laboratory Medicine, Medical University of Gdańsk, 80-210 Gdańsk, Poland; 3 Department of Biology and Cell Imaging, Institute of Zoology and Biomedical Research, Faculty of Biology, Jagiellonian University, 30-387 Kraków, Poland; 4 Department of Laboratory Diagnostics and Microbiology with Blood Bank, Specialist Hospital in Kościerzyna, 83-400 Kościerzyna, Poland; 5 Department of Surgical Oncologic, Medical University of Gdańsk, 80-210 Gdańsk, Poland

**Keywords:** bone cement, nanometals, antibacterial properties, cell viability, hemolysis

## Abstract

Bacterial infections due to bone replacement surgeries require modifications of bone cement with antibacterial components. This study aimed to investigate whether the incorporation of gentamicin or nanometals into bone cement may reduce and to what extent bacterial growth without the loss of overall cytocompatibility and adverse effects in vitro. The bone cement Cemex was used as the base material, modified either with gentamicin sulfate or nanometals: Silver or copper. The inhibition of bacterial adhesion and growth was examined against five different bacterial strains along with integrity of erythrocytes, viability of blood platelets, and dental pulp stem cells. Bone cement modified with nanoAg or nanoCu revealed greater bactericidal effects and prevented the biofilm formation better compared to antibiotic-loaded bone cement. The cement containing nanoAg displayed good cytocompatibility without noticeable hemolysis of erythrocytes or blood platelet disfunction and good viability of dental pulp stem cells (DPSC). On the contrary, the nanoCu cement enhanced hemolysis of erythrocytes, reduced the platelets aggregation, and decreased DPSC viability. Based on these studies, we suggest the modification of bone cement with nanoAg may be a good strategy to provide improved implant fixative for bone regeneration purposes.

## 1. Introduction

Human ageing associated with gradual weakening of the bones and an increasing number of accidents contributes to the fact that bone cement (BC) has been gaining broader applications in medicine. Acrylic BC based on polymethyl methacrylate (PMMA) is a particularly common biomaterial due to its easy processability, favorable mechanical properties, and biostability in the human body. However, it is a non-biodegradable material with relatively poor adhesion to surfaces, and its polymerization can damage the surrounding tissue [1,2,3]. Currently, bone cement is used for the fixation of implants, antibiotic delivery system, cavity or bone defect fillers, coating on metal implants, and vertebral stabilization [4,5,6]. It is generally accepted that clinically successful BC should be a biocompatible material, but some bone cement components may be toxic to human body, such as unreacted methyl methacrylate (MMA) monomer or certain additives, i.e., *N*,*N*-dimethyl-*p*-toluidine, benzoyl peroxide, barium sulfate, as well as free radicals released during the polymerization process. The most recognized negative responses include tissue necrosis, fibrosis, or impaired bone remodeling in the vicinity of the implant [7,8,9]. When introduced into the body, BC may affect blood components, and some BCs used for implantation may affect hemostasis due to the hyperactivation or even inactivation of platelets [10]. The process of platelet activation is essential for maintaining hemostasis and proper wound healing. Activated platelets also release several growth factors, such as TGF-*β*1, PDGF, IGF-I, IGF-II, which are involved in bone remodeling, including osteogenesis, osteoblasts differentiation, and inhibition of osteoclastic bone resorption [10,11]. However, pathological platelet overactivation contributes to the development of micro- and macro-angiopathies leading to vascular complications whereas any BC-related platelet inactivation is associated with the risk of bleeding [12,13]. 

The treatment of complicated fractures with BC is always associated with the opening of the body’s layers. Hence, there is a risk of hospital-acquired infection. Furthermore, the biomaterials (especially BC) handling may lead to the adhesion of bacteria to their surface. It is assumed that the frequency of orthopedic infection is close to 2%, however, for bone substitutes, it has already reached about 13% [14]. Most of these bacterial infections are due to a group of multi-drug resistant clinical bacterial strains, which also produce biofilm. The implant-related osteomyelitis is mainly caused by methicillin-resistant *Staphylococcus aureus* that is exceptionally complicated to cure and usually requires surgical debridement as well as a high dose of locally delivered antibiotics [15,16]. Antibiotic-loaded BC (BC-A) (with commercial or manually added antibiotic) is currently the gold standard that significantly reduces the adhesion and proliferation of bacterial colonies. However, the vast majority of antibiotic particles are released from the BC-A in the first few postoperative hours and thus the dose is too small and ineffective in the next hours or days after implantation. The bacteria may also produce biofilm structures that can significantly reduce the antibiotic’s effects. There is an emerging problem of increased bacterial resistance as well [16,17,18]. Therefore, newer and more effective solutions in implantology are currently sought, including antimicrobial peptides, therapeutic antibodies, phage therapy, quorum sensing inhibitor, as well as antimicrobial nanoparticles [19,20]. Particularly noteworthy are nanometals, such as nanoAu, nanoAg, nanoCu, nanoTi, or nanoZn, which are characterized by high antibacterial abilities and a broad spectrum of activity [19,20,21,22]. BC containing nanometals (BC-N) seem to be a better solution than BC-A, due to better bactericidal properties. The latter are attributed to the release of free metals ions, direct cell membrane damage, uptake of nanoparticles into cells or generating reactive oxygen species (ROS). The above processes may lead to bacteria cell lysis and its mortality, but they may also affect cells of human body [23,24,25]. In general, bacteria are not resistant to nanometals [26,27,28] and nanoAG and nanoCu are now extensively investigated for their antibacterial activity [29,30,31]. These metals are experimentally incorporated into various materials, such as polyurethanes or polypropylene composites, dental adhesives, bioglass, and hydroxyapatite coatings [32,33,34,35,36]. Bone cements can also be enriched with the addition of metal nanoparticles, but so far the investigation of BC containing nanometals or their oxides are scarce. Several studies have been conducted with nanoTiO_2_, nanoMgO, nanoZnO, nanoAl_2_O_3_, nanoAg, and nanoAu to improve mechanical properties of implants or to obtain bactericidal activity but results are often inconclusive [37,38,39,40,41,42,43,44]. The selection of optimal BC modification is thus crucial to provide its safety, protection against infections and effective wound healing in patients [26,45].

In this research, we approach the problem of antibacterial protection along with a good biocompatibility of bone cements by comparing the biological properties of PMMA modified with either gentamycin sulfate or nanoAg and/or nanoCu. So far, very few reports investigated nanoCu modifications of BC and we have not come across the investigations regarding combined nanoAg and nanoCu BC modifications. Antibacterial activity along with erythrocytes integrity, viability of platelets, and dental pulp cells are investigated for the preliminary assessment of modified BC suitability for bone regenerative purposes. 

## 2. Materials and Methods 

### 2.1. Cement Preparation

The PMMA bone cement Cemex (Tecres Company, Verona, Italy) was used as the base material. It was then either modified by us with gentamicin sulfate (Sigma Aldrich, Steinheim, Germany; designated BC-A) or nanometals: Silver or copper (MkNano, Mississauga, ON, Canada; designated BC-N). The latter were designated BC-NpAg, BC-NpCu, BC-NpAg+NpCu. The average particle size of both nanometals was 50 nm, and their purity was 99.9%. The basic properties of nanometals were obtained from the technical sheet. Bone cement specimens were prepared following the manufacturer’s instructions and according to the international standard ISO 5833:2002 [46]. For the modified BCs, the protocol included the addition of modifiers (nanometals or antibiotic) to the cement powder (1.5% or 3% weight/weight) and then manually mixing for about 1 min or up to the visual observation of even distribution of powders. The applied contents of modifiers and bone cement components are presented in Table 1. The used contents of gentamycin and nanometals were set up based on some previous studies [47,48].

### 2.2. Antibacterial Properties Testing on Orthopaedic Bacteria

#### 2.2.1. Bacterial Growth Inhibition 

**Figure 1:**
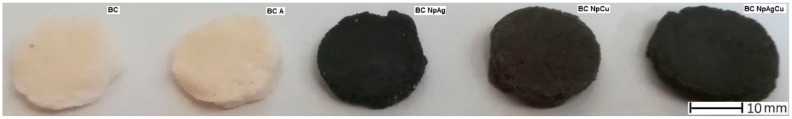
Sample specimens used in the research.

Inhibition of bacterial growth was checked by measuring the turbidity of cultured bacterial broth according to McFarland standards [49]. The study consisted of incubating the tested BCs in a bacterial solution and measuring its optical density. The *Staphylococcus aureus* strain (ATCC 29213) was used for these tests and the initial concentration of bacteria was 1.5 × 10^8^ CFU/mL, which corresponds to 0.5 McFarland index (iMS). The McFarland index is the assumed turbidity of the solution referring to the number of bacteria. Before testing, the BCs were sterilized in an autoclave Sucerex HP 446-1V (Münster Medizin Mechanik, Münster, Germany) at 120 °C for 30 min. The experiment was performed using three specimens for each type of BC (n = 3) in disk form (10 mm diameter, 2 mm thick) and 2 mL of bacterial solution. The size of the specimens was reduced vs. those shown in Figure 1 to adjust samples to the Eppendorf tube size. The assessment of bacterial effectiveness of modifications was carried out on samples incubated with BC specimens with a concentration of 1.5% w/w of modifiers. The bacteria were suspended in Trypticase Soy Broth (Merck, Darmstadt, Germany) and incubated at 37 °C. The DensiChEK Plus (BioMerieux, Montreal, QC, Canada) was used for measurements of optical density of bacteria suspension and readings were made after 0.5, 2, 4, 6, and 24 h. The maximum measuring range of this device is 4 iMS. As control, incubated bacteria suspension without material were used. Following the CLSI Standard M7 [49], the number of bacteria in present tests are calculated based assuming that if iMs equalling 1, the number of bacteria is 3 × 10^8^ CFU/mL, and there is a direct relation between iMS and the number of bacteria. In pursuance of the recommendations for optical densitometry methods, some measurements were rejected as positively false if the material affected the color of the solution.

#### 2.2.2. Inhibition of Bacterial Adhesion to the Surface

Evaluation of bacterial adhesion inhibition was performed by immersing the specimens in a bacterial solution that consisted of five clinically isolated bacterial strains: *Staphylococcus aureus*, *Staphylococcus epidermidis*, *Enterococcus faecalis*, *Enterobacter cloacae*, and *Pseudomonas aeruginosa* (supplied by the Specialized Hospital in Kościerzyna, Poland). A total of 10 mL of each bacterial strains suspensions were taken (inoculum—1 × 10^8^ CFU mL^−1^) and added to 50 mL of the liquid medium—Tryptic Soy Bulion (Merck, Darmstadt, Germany). The experiment was performed using one specimen for BC, BC-A, and BC-NpAg at a concentration of 1.5% w/w. Before the tests, specimens were sterilized in an autoclave at 120 °C for 30 min. The BCs in the form of disks (20 mm diameter, 2 mm thick) were placed in a 2-mL bacterial solution. Then the samples were incubated at 37 °C for 14 days. The adhesion of bacteria to the surface was observed using a scanning electron microscope JSM-7800F (Jeol, Tokyo, Japan).

### 2.3. Cytocompatibility Testing on Blood Components

#### 2.3.1. Blood Collection and Preparation

Red blood cells (RBCs) and platelets (PLTs) were obtained from erythrocyte contaminated buffy coats obtained from the Regional Blood Centre in Gdańsk and provided as by-products of whole blood fractionations according to Regional Blood Blank institutional permission (M-073/17/JJ/11). Whole blood was collected from healthy volunteers in accordance with the Declaration of Helsinki under an approved Regional Bank review board protocol in standard acid citrate dextrose solutions. RBCs and PLTs were fractionated according to standards of Blood Banks [50]. The number of erythrocytes and platelets was estimated with a hemocytometer Superior CE (Marienfeld, Lauda-Königshofen, Germany).

#### 2.3.2. In Vitro Hemolysis Assay and Evaluation of Erythrocyte Morphology

Erythrocytes (3 × 10^9^ cells/mL) were placed in 2 mL tubes containing the autoclaved BC specimens in disk form (10 mm diameter, 2 mm thick) and incubated at 37 °C for up to 24 h. The size of the specimens was reduced vs. those shown in Figure 1 to adjust samples to the Eppendorf tube size. RBCs treated with 2% Triton were used as a positive control (i.e., 100% hemolysis). Briefly, aliquots RBCs exposed to BC specimens for 2 h and 24 h were transferred to microscope slides and the erythrocyte morphology was assessed by light microscopy. Erythrocytes morphology assessment tests were carried out on samples incubated with BC specimens with a concentration of 1.5% w/w. The remaining blood samples were centrifuged at 100× *g* at room temperature for 3 min to let the erythrocytes sediment and supernatants were taken for assessment of hemolysis at a wavelength of 540 nm by Ultrospect 3000pro spectrophotometer (Amersham-Pharmacia-Biotech, Cambridge, UK). The red color of the supernatant indicates damage to RBCs membrane and the intensity of the red color, measured at 540 nm, is assumed as the intensity of hemolysis. According to literature absorbance values which did not exceed the value of 1 were assumed negative for hemolysis [51].

#### 2.3.3. Platelet Aggregation

Platelets (3 × 10^8^/mL) were placed in 2 mL tubes containing the autoclaved BC specimens in disk form (10 mm diameter, 2 mm thick) and preincubated at 37 °C for 2 min and 2 h. This experiment was carried out on BC specimens containing 1.5% of the modifiers additive. After this exposure platelets were transferred to cuvettes for aggregation and resting or thrombin-induced aggregation of platelets (i.e., 0.05 IU) was conducted for 10 min on an aggregometer APACT (Labor, Hamburg, Germany). 

#### 2.3.4. MTT Platelet Viability Test

The analyses of platelet viability were performed by application of thiazolyl blue tetra-zolium bromide (MTT assay; Sigma Aldrich, Steinheim, Germany). Briefly, PLT samples after expose to BC specimens were suspended in phosphate buffered saline (PBS) buffer (Sigma Aldrich, Steinheim, Germany) and plated at a density of 2 × 10^7^ cells. After 4 h of incubation at 37 °C in the dark, a dimethyl sulfide/sodium dodecyl sulfate (Sigma Aldrich, Steinheim, Germany) DMS/SDS solution (20%/3%, pH 4.8) was added to dissolve the reduced formazan product, which reflects cells mitochondrial activity and viability. Finally, the absorbance at 570 (which reflects the blue-violet color for reduced formazan) and 690 nm (which reflects the background from chemicals used in the method) was read in a microplate VICTOR 1420 Multilabel Counter (PerkinElmer, Kraków, Poland) [52]. The color intensity of the solution is proportional to the number of viable cells. The results were compared to control (BC—unmodified bone cement) and assumed as 100% of platelet viability.

### 2.4. Cytocompatibility Testing in Cultures of Dental Pulp Stem Cells (DPSC)

#### 2.4.1. DPSC Collection and Preparation

Bone cements are widely used in both orthopedic and dental surgeries [2,15,53]. We chose human dental pulp stem cells (DPSC), which are routinely obtained in one of our laboratories and they are a good model of adult stem cells with great potential to regenerate bone [54]. DPSC were obtained from the molar tooth of an adult 31-year-old female donor in agreement with the Polish Research Ethics Board; approval no. 1072.6120.253.2017). The cells were isolated according to a *Bakkar protocol* (2017) [55], expanded in culture using alpha-MEM supplemented with 15% fetal bovine serum (FBS), 0.1 mM l-ascorbic acid phosphate and 1% antibiotics (Penicilin-Streptomycin—10,000 U/mL, Thermo Fisher Scientific, Waltham, MA, USA). When the cells reached a confluent monolayer, they were lifted from culture flasks using 0.25% Trypsin-EDTA, counted and seeded directly on the surface of the materials. The cells were then used for preliminary assessment of their proliferation potential, adhesion, and morphology on BC surfaces. Briefly, the cell culture experiments were performed using three specimens for each type of BC (n = 3) in disk form (20 mm diameter, 2 mm thick). Before testing, the specimens were sterilized by autoclaving at 120 °C for 30 min. The specimens were then placed in separate wells of 24-well culture plates and covered by 2 × 10^4^ DPSC suspended in 1 mL of culture medium (i.e., alpha-MEM supplemented with 10% fetal bovine serum (FBS) and antibiotics). The cells were incubated at 37 °C in an atmosphere containing 5% CO_2_ for up to 5 days. The medium was aspirated and replaced with a fresh one every 48 h.

#### 2.4.2. MTS Cell Viability Test

DPSC were tested for viability after 4 days of culture. Cell viability was evaluated using CellTiter 96 Aqueous One Solution Cell Proliferation Assay (MTS, Promega, Kraków, Poland). Each material sample in the culture plate was covered with 400 µL of MTS solution diluted ten times in phenol-red free alpha-MEM. The plate was then incubated at 37 °C in a culture incubator until the development of a brownish color of the MTS solution. The intensity of the developed color is proportional to the number of actively metabolizing live cells. After 30 min, the MTS solution from individual wells was transferred to clear 96-well plates, and its absorbance was measured at 490 nm, where is the maximum absorbance of the solution. The results were expressed as a % change in the live cell number compared to the results obtained for cells grown on BC (assumed as 100%).

#### 2.4.3. Evaluation of DPSC Cells Morphology 

To evaluate the potential morphological changes of cells on contact with the materials, we used larger culture wells (12-well plates) and a higher DPSC cell suspension was applied for this study (i.e., 4 × 10^4^ cells suspended in 2 mL of medium). The morphology of DPSC cells in close proximity to the materials was observed with contrast-phase inverse microscope Axiovert 40 CFL (Zeiss, Oberkochen, Germany) at culture days 1, 3, and 5. For control, the cells were also seeded into standard culture plates without any specimens. Photographs were taken for DPSC samples cultured on BC specimens with a concentration of 3% w/w of modifiers.

#### 2.4.4. Adhesion Assessment of DPSC Cells to the Surface 

Cell adhesion to the BC surface was evaluated after 24 h incubation of BC specimens with DPSC cells using fluorescence microscope, contrast-phase inverse microscope Axiovert 40 CFL (Zeiss, Oberkochen, Germany), and scanning electron microscope JSM-7800F (Jeol, Tokyo, Japan). For these analyses cells were seeded directly on materials in the form of disks (20 mm diameter, 2 mm thick) in separate wells of 24-well culture plates a the density of 2 × 10^4^ DPSC in 1 mL of culture medium. After 24 h incubation cells were either directly observed under contrast-phase microscope or fixed and/or stained for SEM or fluorescence microscope observations, respectively. These experiments were carried out on samples cultured with BC and BC-NpAg at 3% w/w. For fluorescent microscopy, cell cultures on material specimens were fixed with a 4% solution of formaldehyde (Sigma Aldrich, Steinheim, Germany) and incubated for 30 min at 37 °C. After washing three times with PBS solution, 0.03% Evans blue stain solution (Sigma Aldrich, Steinheim, Germany) was added for 20 min followed by rinsing three times with PBS solution to remove excess strain [56]. For SEM analyses cultures were fixed with a solution composed of 2% formaldehyde, 2% glutaraldehyde, and 2% cacodyl buffer (Sigma Aldrich, Steinheim, Germany) for 24 h at 4 °C. Next the fixed cultures were rinsed three times with PBS and dehydrated in increasing concentrations of ethyl alcohol (Sigma Aldrich, Steinheim, Germany) for 30 min [57].

### 2.5. Statistical Method 

Statistical analysis of the data was performed using commercial software (SigmaPlot 14.0, Systat Software, San Jose, CA, USA). The Shapiro–Wilk test was used to assess the normal distribution of the data. All of the results were presented as a mean ± standard deviation (SD) and were statistically analyzed using one-way analysis of variance (one-way ANOVA). Multiple comparisons versus the control group between means were performed using the Bonferroni *t*-test with the statistical significance set at *p* < 0.05.

## 3. Results

### 3.1. Antibacterial Properties Testing on Orthopedic Bacteria

#### 3.1.1. Bacterial Growth Inhibition

The assessment of the inhibition of bacterial growth in solution at its initial stage was carried out. In the case of bacteria incubated in the control sample and with BC, their rapid multiplication to 4 iMS (which corresponds to 12 × 10^8^ CFU/mL) was observed within 4 h. In the case of modified BC, the growth of bacteria was slowed down. After 24 h, the turbidity value of the solution was 1.33 iMS for BC-NpAg (about 3.9 × 10^8^ CFU/mL; turbidity about 93.1 NTU), 1.52 iMS for BC-A (about 4.6 × 10^8^ CFU/mL; turbidity about 119.4 NTU), and 0.91 iMS for BC-NpAg+Cu (about 2.7 × 10^8^ CFU/mL; turbidity about 60.2 NTU). Hence, a significant inhibition of bacterial growth in the initial incubation phase with the material is confirmed (Table 2).

#### 3.1.2. Inhibition of Adhesion of Bacteria to the Surface

**Figure 2:**
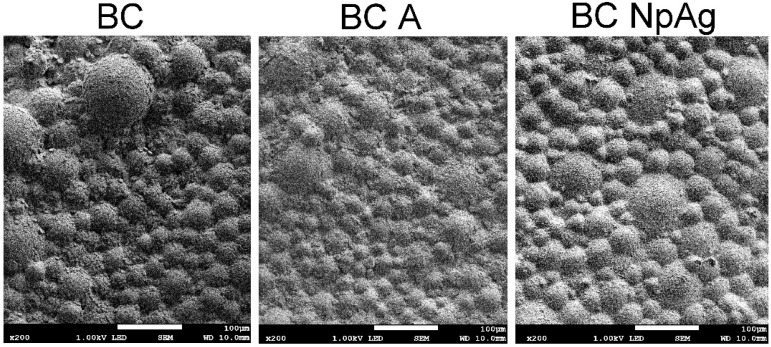
Comparison of bacterial adhesion to the surface of the tested materials after 14 days of incubation in bacterial suspension: Modified bone cements—1.5% w/w of modifiers additive (SEM 200×).

The long-term antibacterial effect was checked by bacterial adhesion to the surface of BCs after 14 days. In the case of BC-A, a decrease in the bacteria staked to the surface was observed. However, even better effects were observed for BC-NpAg (Figure 2). Additionally, on the surface of BC, formation of the biofilm structure appeared (Figure 2).

### 3.2. Cytocompatibility of Bioactive Bone Cements with Erythrocytes and Blood Platelets 

#### 3.2.1. Effect of Bone Cement Modifications on In Vitro Hemolysis and Erythrocyte Morphology 

**Figure 3:**
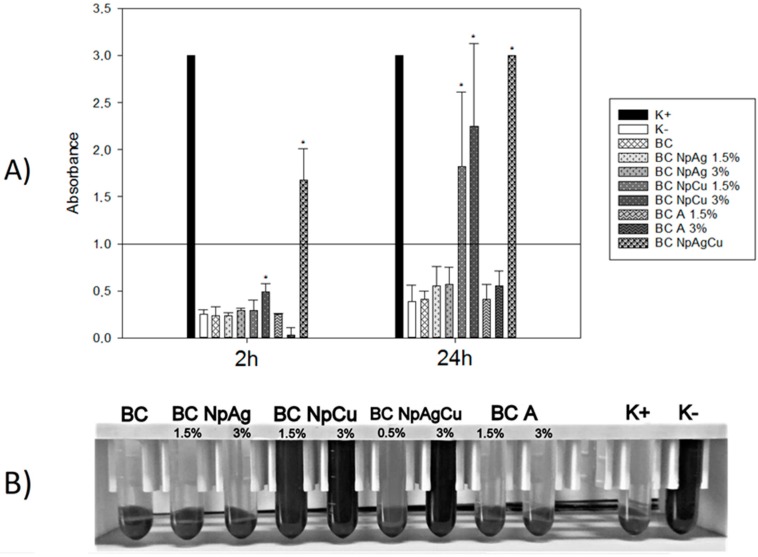
Erythrocyte hemolysis after in vitro exposure to unmodified and modified cement specimens: (**A**) Hemolysis at 2 h and 24 h incubation of materials with erythrocytes—the line indicates the threshold value above which the hemolysis take place (n = 5; data are the means ± SD; * significantly different from negative control—*p* < 0.05); (**B**) Hemolysis after 24 h incubation of specimens with erythrocytes (the presented pictures are representative for 5 experiments).

**Figure 4:**
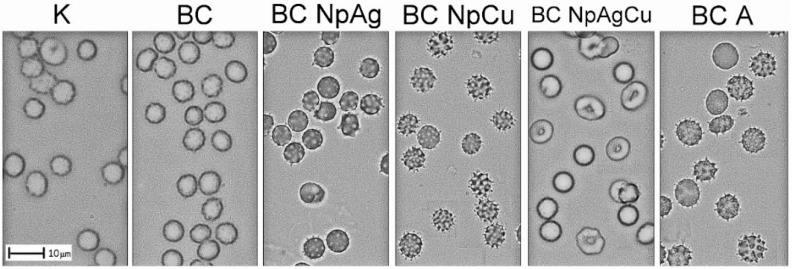
Morphology of RBCSs exposed to cement specimens for 24 h: Modified bone cements with 1.5% w/w of modifiers (the presented pictures are representative for 5 experiments).

As presented in Figure 3, after 2 h exposure of RBCs to BC, 1.5% and 3% BC-NpAg, 1.5% and 3% BC-NpCu, and 1.5% and 3% BC-A hemolysis of erythrocytes was not observed expect for erythrocytes incubated with BC-NpAg+Cu (*p* < 0.05). After 24 h of RBCs incubation with the specimens, there was significant hemolysis in RBCs exposed to 1.5% and 3% BC-NpCu as well as BC-Ag+Cu (*p* < 0.05). The morphology of erythrocytes was assessed after a longer incubation with specimens (24 h) (Figure 4). There was no anisocytosis of RBCs exposed to the BC specimens. However, there was evidence of RBCs poikilocytosis after incubation with: BC-NpAg 1.5% and 3%, BC-NpCu 1.5% and 3%, BC-A 1.5% and 3%, as well as BC-NpAg+Cu. After 24 h incubation with the specimens containing 1.5% and 3% nanoAg, 1.5% and 3% nanoCu, and 1.5% and 3% gentamicin evoked RBCs membrane shape changed similar to echinocytes or acanthocytes. Interestingly, the combination of both nanometals Ag and Cu (1.5%) modified RBCs shape differently and similar to codocytes or target cells.

#### 3.2.2. Effect of Bone Cement Modifications on In Vitro Platelet Aggregation and Their Viability

There was no spontaneous platelets aggregation after 2 min and 2 h exposure to BC, BC-NpAg 1.5%, BC-NpCu 1.5%, and BC-A 1.5% (Table 3). Short (2 min) exposure of PLTs to BC, BC-NpAg, BC-NpCu, and BC-A did not significantly change both the early phase and the late phase of thrombin-evoked aggregation (Table 3). However, longer (2 h) PLTs incubation with BC-NpCu 1.5% reduced the early phase of aggregation by 87% and the late phase of aggregation by 67% (*p* < 0.05) (Table 3). Similarly, BC-A 1.5% reduced thrombin-induced platelets aggregation by 67% and 41% in the early phase and the late phase (*p* < 0.05) (Table 3). Moreover, an MTT test was performed after 2 h incubation specimens with platelets. A significant reduction was found only in the case of BC-NpCu (about 60% compared to the control; *p* < 0.05; data unpublished).

### 3.3. Cytocompatibility of Bioactive Bone Cement with Dental Pulp Stem Cells 

#### 3.3.1. MTS Cell Viability Test 

**Figure 5:**
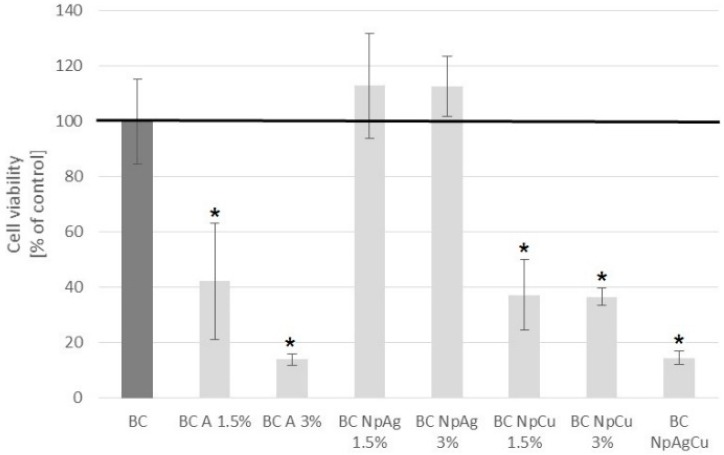
Dental pulp stem cell (DPSC) viability on modified bone cements at day 4 culture. Results are expressed as % change in cell viability compared to the unmodified bone cement (n = 3; mean ± SD; * significantly different from unmodified bone cement—*p* < 0.05).

The viability of dental pulp stem cells (DPSCs) after 3 days culture on material specimens, is presented in Figure 5. 

BC 1.5% and 3% NpAg showed similar to BC cell viability and there was no statistical difference cells grown (Figure 5). However, the other modifications (BC-A, BC-NpCu, as well as BC-NpAg+Cu) significantly decreased cell viability compared to cells grown on BC (Figure 5).

#### 3.3.2. Evaluation of DPSC Cells Morphology

**Figure 6:**
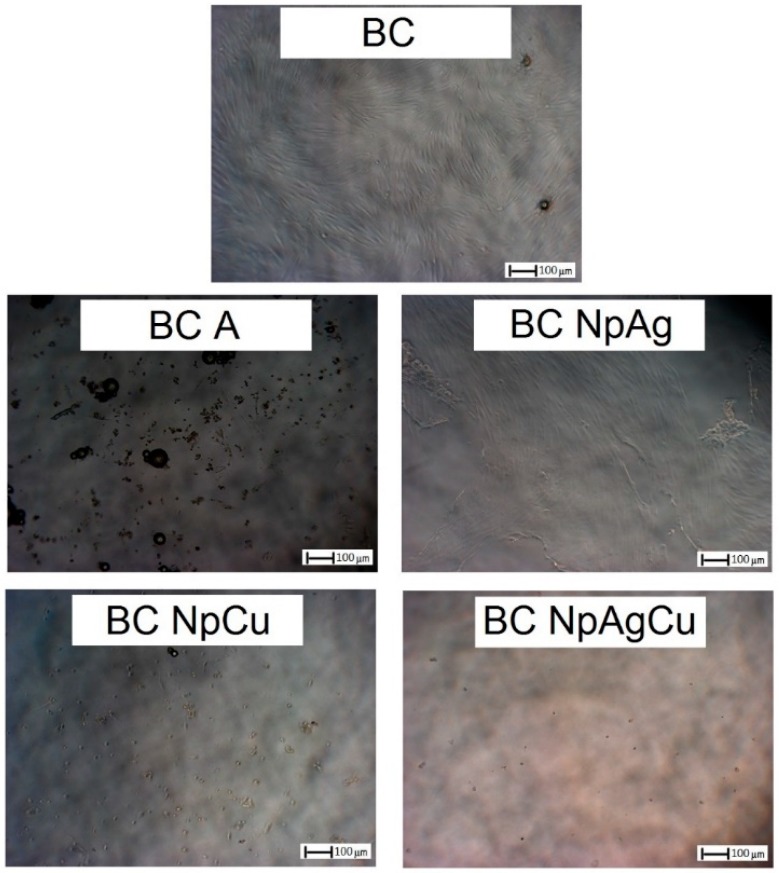
Evaluation of morphology of cells grown in close proximity to the materials: Modified bone cements with 3% w/w of modifiers (the presented pictures are representative for 3 experiments).

The morphology of cells grown in close proximity to the materials compared to normal cell morphology grown on tissue culture plastic (TCP) was evaluated. Photographs taken under a contrast-phase inverse microscope are shown in Figure 6. Unmodified BC and BC-NpAg did not affect the DPSC morphology and confluent monolayer could be observed in the proximity of materials (Figure 6). In contrast, toxicity of nanoCu was found as we observed reduced number of cells and they displayed abnormal, rounded morphology (Figure 6). It may be either due to the inability of cells to adhere or cell detachment due to material dissolution products.

#### 3.3.3. Adhesion Assessment of DPSC Cells to the Material Surfaces

**Figure 7:**
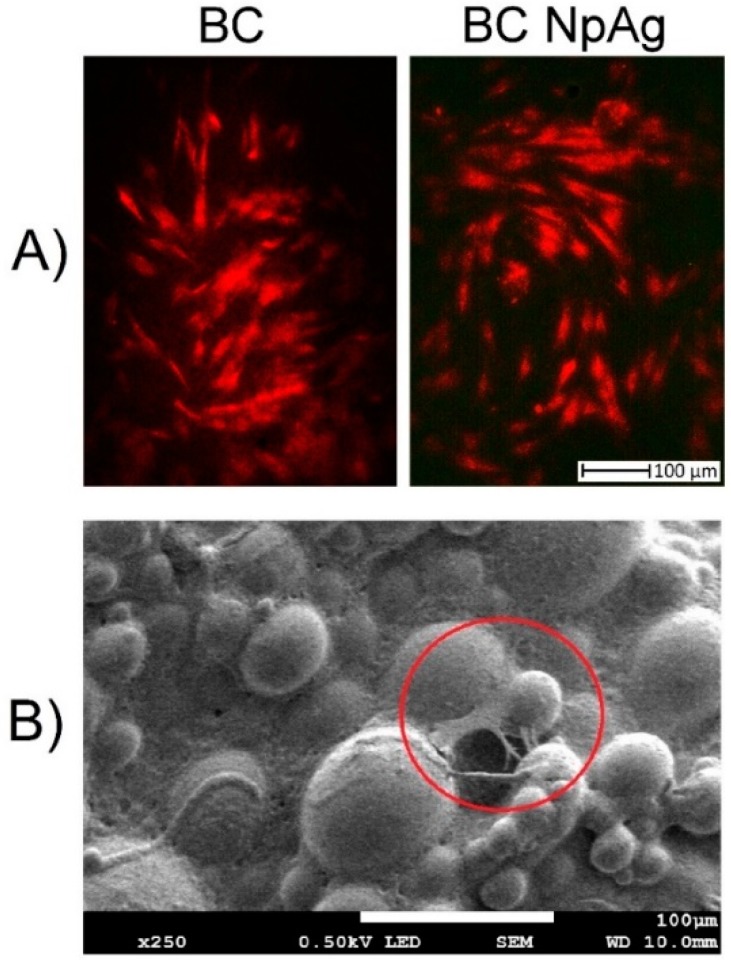
DPSC adhesion to the surface of bone cement (BC) and BC containing nanoAg (BC NpAg) (3% w/w) after 24 h (the presented pictures are representative for 3 experiments): (**A**) Images from a fluorescence microscope, Evans blue straining of cells frown on BC and BC-NpAg; (**B**) images from a scanning electron microscope for BC-NpAg showing adhered cells marked by red circle.

Twenty-four hours post cell seeding cells adhered well to BC and BC-NPAg as shown by Figure 7A staining with Evans Blue and observation with fluorescence microscopy as well as by Figure 7B fixing and observation with scanning electron microscopy. For all other material modifications, only single cells were found on the surface.

## 4. Discussion

Pure bone cement is approved by the U.S. Food and Drug Administration for clinical purposes [58]. However, its modification such as additives (e.g., nanometals or antibiotics) can cause adverse effects. Therefore, the cytocompatibility of BC containing gentamicin, nanoAg, nanoCu, or nanometals combination was assessed in direct contact with human blood cells and dental pulp stem cells. The presented data confirmed the cytocompatibility of the commercially available BC after a short and a long-term exposure to both blood cells and DPSC cells (Figure 3, Figure 4, Figure 5, Figure 6 and Figure 7, Table 3). These results are consistent with previous reports that pure BC does not cause blood hemolysis or reduces the number of platelets or osteocyte-like cells [10,59]. Similar results to those presented in this manuscript were obtained for BC-A enriched with additives (i.e., gentamicin, vancomycin, or ciprofloxacin) [15,16,17,60]. However, we show that the exposure of DPSC cells to BCs containing gentamicin (1.5% w/w) decreased their viability by 60% (Figure 5). It may be due to the excessive level of antibiotics as the culture medium for DPSC is routinely supplemented with penicillin and streptomycin and on top of that, it was enriched with specimens released from the BC. Thus, the cells might have been exposed to a toxic dose of antibiotics [61]. It is a significant result indicating the potentially undesired effect of combinations of antibiotics in the growth of osteoblastic progenitors.

The addition of nanoAg additives to BC (up to 3% w/w) did not affect the viability of blood cells and DPSC cells and their antibacterial function (Figure 3, Figure 4, Figure 5, Figure 6 and Figure 7, Table 3). These findings correspond to previous reports demonstrating that BC with added silver is not harmful to different cell lines, including mouse osteoblast TMOb cells (nanoAg 30–50 nm; 0.25%, 0.5%, and 1.0% w/w, after 3 days) [62], mouse preosteoblast MC-3T3 cells (nanoAg 5 nm or 11 nm; 0.01%, 0.05%, and 1.0% w/w, after 2 days) [63,64], human fetal osteoblast hFOB cells (nanoAg 5–50 nm; 0.1%, 0.5%, and 1.0% w/w, after 2 days) [65], and human mesenchymal stem MSC cells (nanoAg 4000 µg/g) [66]. On the other side, the toxicity of nanoAg was reported by other researchers. For example, BC-NpAg caused hemolysis of erythrocytes and reduced the hBMSCs cell viability [66,67,68]. It should be noted that in earlier studies the routine concentration of nanoAg was up to 1%, much lower compared to the levels studies here. Despite that, no undesired impacts of nanoAg on the survival of blood cells and DPSC cells were observed in our present study (Figure 3, Figure 4, Figure 5, Figure 6 and Figure 7, Table 3). BC-NpAg up to 3% did not induce any hemolysis except it changed the morphology of erythrocytes after a longer 24 h exposure; it did not affect the DPSC cell viability, their morphology, and the cells’ adhesion, and also it did not inhibit blood platelet function (Figure 3, Figure 4, Figure 5, Figure 6 and Figure 7, Table 3). Such discrepancy between these and previous studies may be associated with either feature of the nanoAg itself (size or purity) or the research methodology used by the other researchers compared with these studies. The changes in the erythrocytes’ shape in the present research may be directly associated with long-term incubation or handling of blood (Figure 4). Determining whether these changes are reversible and toxic requires further investigations. Surprisingly, in contrast to nanoAg, the use of the nanoCu additives (1.5% and 3%, alone or with nanoAg) reduces blood cells and DPSC cells viability as well as affects their morphology (Figure 3, Figure 4, Figure 5 and Figure 6, Table 3). To our knowledge, the effects of nanoCu enriched BC on its biocompatibility and cytotoxicity have not been extensively studied. The acute toxicity of nanoCu was reported to damage the liver, the kidney, and the spleen [69,70], and the use of nanoCu beyond the safety limits (depending on many factors) would lead to severe cytotoxicity [71,72]. With regards to the cytocompatibility, the data on biopolymers containing Cu are scarce. The report on nanoCu/low-density polyethylene nanocomposite intrauterine device indicated the possibility of using nanoCu without systemic toxicity [73]. Furthermore, in the study with no nanometric Cu, the growth of pre-osteoblast cells and their differentiation was observed [72,74]. Moreover, Cu doped bioactive glass scaffold (up to 5% w/w) demonstrated no adverse effect on the viability of DPSC or BMSC cells and enhancing osteogenesis [34,74,75]. Different results related to toxicity obtained in this study may be attributed to the use of Cu of nanometer size (Figure 3, Figure 4, Figure 5 and Figure 6, Table 3), potentially more dangerous than other nanoparticles. In similar studies, Cu doped bioglass caused toxicity of human bone osteosarcoma HOS cells (2.5% w/w) [33] and human osteosarcoma SaOS-2 cells (5% or 10% w/w) [76]. There are no reports so far on the combination of nanometals, such as nanoAg and nanoCu, as in the present study. The obtained results demonstrated that both elements, when added together, provoke cytotoxicity in BCs (Figure 3, Figure 4, Figure 5 and Figure 6, Table 3). This means that nanoCu, either added alone or combined with nanoAg, is harmful to cells and cannot be recommended. Some studies on copper toxicity report that the toxic effects of Cu/nanoCu are mainly associated with ROS production, but also they can be associated with the damage to the membrane, electron transport disturbance, DNA damage, and results in developmental abnormalities [77]. 

Pure BC has no bactericidal properties, but also may contribute to infections. The use of antibacterial additives (mainly antibiotics) allows prevention of bacteria growth on the BC [15,16]. Therefore, bactericidal properties of BC containing gentamicin (BC-A), nanoAg (BC-NpAg), nanoCu (BC-NpCu), or nanometals (BC-NpAg+Cu) combination were assessed. This research confirmed the bactericidal properties of all modified BCs (Figure 2, Table 2), and nanoAg was more effective than gentamicin (Figure 2, Table 2). Those findings are consistent with previous research demonstrating that BC or PMMA coating/film containing Ag were effective against different bacteria strains including *S. aureus* (5–50 nm; 0.25%, 0.5%, and 1.0% w/w after 24 h) [44,62], *S. epidermidis* (5–50 nm; 0.25%, 0.5%, and 1.0% w/w after 24–48 h) [44,45,62,63,64], *A. baumanni* (5 nm; 0.25%, 0.5%, and 1.0% w/w after 24 h) [62], MRSA (5–10 nm; 0.25%, 0.5%, and 1.0% w/w after 24 h) [62,63], *P. aeruginosa* (5–10nm; 0.1%, and 1.0% w/w after 16 h) [21], *E. coli* (20-27 nm; after 18 h) [78]. In most of the above studies, it was found that the appropriate concentration of nanoAg was 1% w/w, which is in line with present results (1.5% w/w of nanoAg) (Table 2). The antibacterial effectiveness increased with the increasing content of additives. Our observations (Figure 2) are in part supported by past research showing the reduction of biofilm formation on the surface of the modified material [21,42,61]. The obtained results confirmed then the superiority of nanoAg over gentamicin (Figure 2, Table 2). Some reports suggested the ineffectiveness of BC-NpAg (5–50 nm; concentration 0.25–1% w/w) [44,79]. These discrepancies may be due to the research methodology and a weak release of nanoAg from the BC or its non-ionized form. However, in our current and previous studies the effectiveness of nanoAg in preventing bacteria adhesion as well as combating surrounding bacteria was demonstrated (Figure 2, Table 2) [47]. The BCs containing nanoCu or both nanometals have not been investigated yet. Some investigations of nanoCu in polymer matrices and its bactericidal properties are consistent with the present results (Table 2) [80,81,82]. 

The bactericidal effectiveness, as well as the toxic effect of nanometals, depends on its type, dose, size, total surface area, as well as agglomeration [65,83]. In this research, two nanometals (nanoAg and nanoCu) of equal size were used, and both showed bactericidal properties, but two different types of cells response were observed. Hence, it can be concluded that the type of nanometals affects cytotoxicity. Furthermore, the recent research has resulted in enlargement of Ag-based nanostructures for potential medical and other application. This includes silver nanospheres that are created to kill effectively a variety of bacterial and fungal strains [84]. Another example is AlOOH–Ag nanocomposite applied against both Gram-negative and Gram-positive microorganisms in catalysis, water purification, and biomedical applications [85]. Yet another antimicrobial nanoAg-TiO2 coating was applied for lining leather and proved effective to prevent growth of four bacterial strains [86]. It remains unresolved why the same dose of nanoparticles can be cytotoxic to eukaryotic cells and not to prokaryotic cells. It is plausible this is related to the cell organization of prokaryotic and eukaryotic cells. Firstly, eukaryotic cells are usually bigger and have higher structural and functional redundancy. Secondly, they contain several organelles such as mitochondrion, cell nucleus, Golgi apparatus. or endoplasmic reticulum, and these are additional diffusion barriers for nanoparticles. Thirdly, eukaryotic cells usually contain more than one mitochondrion. Therefore, it is much more difficult for nanometals or their ions to cause eukaryotic cell death [61,87,88]. The above explanations justify why the applied nanoAg modification of BC is bactericidal, but it is not toxic to cells. 

The present study has some limitations which indicate directions of further research. At first, the here applied nanometals with a particle size of 50 nm were the compromise between bactericidal effectiveness which would likely be better for smaller nanoparticles, but they would be presumably more toxic. Secondly, this study focused exclusively on the antibacterial biological properties of BC, and based on the obtained results, the chemical, physical, and mechanical properties of biologically best and non-toxic BCs will be investigated soon. Thirdly, the present investigations of cytocompatibility and bactericidal effectiveness were carried out using short-term exposure in in vitro conditions and further research will include long-term exposure and/or in vivo tests, including additional environmental factors, i.e., anionic ligands (such as: Chloride, inorganic sulfide) and/or proteins appearing in biological media that may bind metallic ions or even nanometals that may be eliminated by the immune system [65,66,89]. Finally, a thorough assessment of long-term nanometals and biological effectiveness will be carried out.

## 5. Conclusions

In this study, commercially available acrylic bone cement was modified with gentamicin, nanosilver, nanocopper, or their combination to obtain increased bactericidal properties while not deteriorating cytocompatibility. The obtained results demonstrate that cement modified with 1.5% and 3% w/w nanoAg or nanoCu shows a higher bactericidal effect and antibiofilm properties compared with antibiotic-loaded bone cement. Furthermore, cement containing nanoAg (1.5% and 3% w/w) does not influence erythrocytes hemolysis, blood platelet function, viability, morphology, and adhesion of dental pulp stem cells. On the other hand, cement containing nanoCu (1.5% and 3% w/w) induces a shape change and the hemolysis of erythrocytes, reduces platelet aggregation, and decreases the viability of dental pulp stem cells. The observed differences in effects of nanoAg and nanoCu, positive and negative, may be very specific and due to nanometric size and direct interaction of such nanoparticles with cells or their modification of biochemical reactions. Bone cement modified with nanoAg may be an alternative option for future clinical applications after further biological research to fully confirm their biomechanical safety and biocompatibility, and after all the tests on their chemical, mechanical, and physical properties are completed.

## Figures and Tables

**Table 1:** Chemical composition of bone cements used for the research.

Chemical Composition	Unmodified Bone Cement/BC/	Antibiotic-Loaded Bone Cement/BC A/		Bone Cement Modified with Nanometals					
				nanoAg/BC-NpAg/		nanoCu/BC-NpCu/		nanoAg & nanoCu/BC-NpAg+Cu/	
**Powder Component (% w/w)**									
Polymethyl methacrylate	84.30	83.05	81.77	83.05	81.77	83.05	81.77	83.05	
Barium sulfate	13.00	12.80	12.61	12.80	12.61	12.80	12.61	12.80	
Benzoyl peroxide	2.70	2.65	2.62	2.65	2.62	2.65	2.62	2.65	
Gentamicin sulphate	-------	1.50	3.00	-------	-------	-------	-------	-------	
NanoAg	-------	-------	------	1.50	3.00	-------	-------	1.15	1.50
NanoCu	-------	-------	------	-------	------	1.50	3.00	0.35	
**Liquid Component (% w/w)**									
Methyl Methacrylate	99.10								
*N*,*N*-dimethyl-*p*-toluidine	0.90								
Hydroquinone	75.00								

**Table 2:** McFarland standard values specifying the number of *Staphylococcus aureus* bacteria during incubation with tested specimens: Modified bone cements—1.5% w/w of modifiers additive (n = 3; mean ± SD; * significantly different from control—*p* < 0.05).

McFarland Index						
Time (h)	K	BC	BC-NpAg	BC-NpCu	BC-NpAg+Cu	BC-A
0	0.5 ± 0.01					
0.5	0.68 ± 0.01	0.71 ± 0.02	0.68 ± 0.01	0.70 ± 0.02	0.60 ± 0.01	0.65 ± 0.01
2	1.93 ± 0.03	1.64 ± 0.01	1.10 ± 0.01	1.95 ± 0.01	0.77 ± 0.02	1.24 ± 0.01
4	>4	3.48 ± 0.02	1.22 ± 0.02	×	0.79 ± 0.02	1.35 ± 0.02
6	>4	>4	1.29 ± 0.01	×	0.80 ± 0.03	1.48 ± 0.02
24	>4	>4	1.33 ± 0.02 *	×	0.91 ± 0.03 *	1.52 ± 0.02 *

* Statistical analysis was performed between groups and control after 24 h and the group, where the statistically significant difference occurred was marked. ×—measurement rejected due to the color of the solution.

**Table 3:** Effect of cement specimens on platelet aggregation: Modified bone cements with 1.5% w/w of modifiers/(n = 5; data are the means ± SD; * significantly different from negative control—*p* < 0.05).

	K		BC		BC-NpAg		BC-NpCu		BC-A	
	2′	2 h	2′	2 h	2′	2 h	2′	2 h	2′	2 h
Spontaneous aggregation (%)	1 ± 1	2 ± 1	1 ± 1	2 ± 1	2 ± 1	2 ± 1	2 ± 1	2 ± 1	1 ± 1	2 ± 1
Trombin-evoked early phase of aggregation (1 min) (%)	43 ± 6	31 ± 8	39 ± 3	23 ± 11	33 ± 8	23 ± 6	28 ± 13	4 ± 2 *	32 ± 14	10 ± 3 *
Thrombin-evoked late phase of aggregation (10 min) (%)	76 ± 9	67 ± 7	74 ± 8	64 ± 10	70 ± 8	49 ± 9	69 ± 6	22 ± 17 *	67 ± 13	39 ± 7 *
